# Accumulation of Phosphorus-Containing Compounds in Developing Seeds of Low-Phytate Pea (*Pisum sativum* L.) Mutants

**DOI:** 10.3390/plants4010001

**Published:** 2014-12-26

**Authors:** Arun S.K. Shunmugam, Cheryl Bock, Gene C. Arganosa, Fawzy Georges, Gordon R. Gray, Thomas D. Warkentin

**Affiliations:** 1 Crop Development Centre, Department of Plant Sciences, University of Saskatchewan, 51 Campus Drive, Saskatoon, SK S7N 5A8, Canada; E-Mails: s.ask@usask.ca (A.S.K.S.); gene.arganosa@usask.ca (G.C.A.); gr.gray@usask.ca (G.R.G.); 2 National Research Council Canada, 110 Gymnasium Place, Saskatoon, SK S7N 0W9, Canada; E-Mails: Cheryl.bock@nrc-cnrc.gc.ca (C.B.); fawzy.georges@usask.ca (F.G.)

**Keywords:** D-*myo*-inositol phosphate synthase (MIPS), phosphorus, phytate biosynthesis, phytic acid

## Abstract

Low phytic acid (*lpa*) crops are low in phytic acid and high in inorganic phosphorus (P_i_). In this study, two *lpa* pea genotypes, 1-150-81, 1-2347-144, and their progenitor CDC Bronco were grown in field trials for two years. The *lpa* genotypes were lower in IP_6_ and higher in Pi when compared to CDC Bronco. The total P concentration was similar in *lpa* genotypes and CDC Bronco throughout the seed development. The action of *myo*-inositol phosphate synthase (MIPS) (EC 5.5.1.4) is the first and rate-limiting step in the phytic acid biosynthesis pathway. Aiming at understanding the genetic basis of the *lpa* mutation in the pea, a 1530 bp open reading frame of *MIPS* was amplified from CDC Bronco and the *lpa* genotypes. Sequencing results showed no difference in coding sequence in *MIPS* between CDC Bronco and *lpa* genotypes. Transcription levels of MIPS were relatively lower at 49 days after flowering (DAF) than at 14 DAF for CDC Bronco and *lpa* lines. This study elucidated the rate and accumulation of phosphorus compounds in *lpa* genotypes. The data also demonstrated that mutation in *MIPS* was not responsible for the *lpa* trait in these pea lines.

## 1. Introduction

Phytic acid (*myo*-inositol-1,2,3,4,5,6-hexa*kis*phosphate; IP_6_) is the major storage form of phosphorus (P) in most plant seeds [1]. Phytate is present within subcellular protein inclusions in all seeds. Particularly, in cereal grains, phytates are essentially localized in the germ and aleurone tissues whereas in dicotyledons, phytate is distributed throughout the cotyledon [2,3]. Endogenous phytase enzymes break down phytate during seed germination and release its phosphorus, *myo*-inositol, and mineral contents for use by the growing seedling [4]. IP_6_ also accumulates in other plant tissues and organs that accumulate nutrient stores for subsequent redistribution, such as pollen, roots, and tubers [5].

Applied interest in seed IP_6_ primarily concerns its roles in human health and animal nutrition. It is a strong chelator of mineral elements such as iron, zinc, calcium, and potassium, forming mixed salts that are largely excreted by humans and other non-ruminant animals such as poultry, swine, and fish [6,7]. Excretion of seed-derived IP_6_ can contribute to dietary iron and zinc deficiencies, a major public health problem in the developing world. In addition, undigested phytate excreted by non-ruminant animals represents an important source of phosphorus pollution in the environment [8]. Due to nutritional and environmental concerns, the development of cultivars with a low-phytate trait has become an attractive breeding objective in many crop species. Chemically induced, non-lethal recessive mutants that decrease seed phytic acid content have been isolated and genetically mapped in maize (*Zea mays* L.) [9,10], barley (*Hordeum vulgare* L.) [11,12], and soybean (*Glycine max* L. Merr) [13]. Recently, Warkentin *et al.* [14] developed and characterized two low-phytic acid mutants of field pea (*Pisum sativum* L.). The low phytic acid (*lpa*) mutations have the potential to alleviate the environmental and nutritional problems associated with phytic acid in animal feeds [15]. Moreover, *lpa* crops may also offer improved nutrition for human populations that depend upon grains and legumes as staple foods. In addition, these *lpa* mutants provide a valuable system to study seed phytic acid synthesis.

In plants, the six-carbon cyclitol *myo*-inositol gives rise to compounds with roles in such diverse functions as signal transduction, membrane biogenesis, stress tolerance, and the generation of seed storage compounds including IP_6_ [16,17]. The *de novo* synthesis of *myo*-inositol involves the conversion of glucose 6-phosphate to *myo*-inositol-1-phosphate (IP_1_) that is subsequently dephosphorylated to release free *myo*-inositol. The former reaction is catalyzed by *myo*-inositol-1-phosphate synthase (MIPS; EC 5.5.1.4), the first and rate-limiting enzyme of the pathway [17,18,19]. This makes *MIPS* an attractive target for manipulation to produce low-phytate crops. Previous studies have demonstrated that 50% to 95% reductions in phytic acid can be obtained when this enzyme is targeted through mutagenesis or by genetic engineering methodologies [20,21,22,23,24,25,26]. In soybean, mutations in *MIPS* coding sequences conferred a decreased phytic acid phenotype, and an effective reduction in phytate content (90% to 95%) has been observed when one of the four *MIPS* in soybean, *GmMIPS1* was silenced through a RNA interference (RNAi) approach [24,26]. An *lpa* phenotype was also produced by manipulating the *MIPS* gene through an antisense approach in *Oryza sativa* L. [22].

The objective of this study was to investigate the accumulation of phytic acid and other phosphorus compounds in developing seeds of normal and low-phytate genotypes of field pea (*Pisum sativum* L.). We also examined *MIPS* gene expression and analyzed the sequence at the nucleotide and protein levels to ascertain if variation in *MIPS* coding sequences was responsible for the *lpa* trait. This will help us to understand the nature of the low phytate mutation(s) and develop markers for the low phytate trait furthering the development of low-phytate cultivars.

## 2. Results

### 2.1. Agronomic Characteristics of Low-Phytate Pea Genotypes

**Figure 1:**
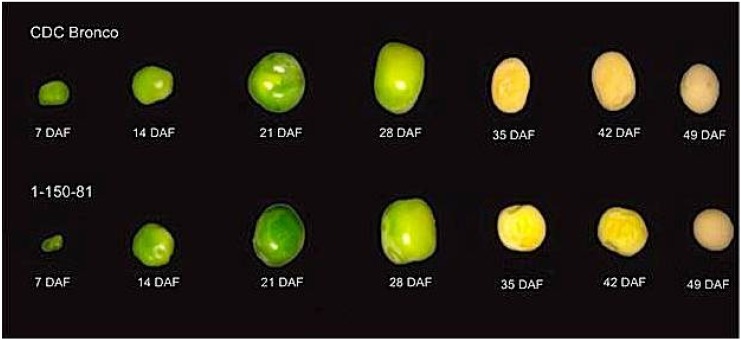
Developing seeds of pea (*Pisum sativum* L.). Indicated are CDC Bronco and low-phytate genotype 1-150-81. Representative photographs are shown from the Rosthern site in 2010. DAF stands for “days after flowering”.

The agronomic characteristics of the *lpa* pea genotypes were similar to their normal phytate progenitor CDC Bronco except for 1000 seed weight and grain yield at maturity (Figure 1, Table 1). CDC Bronco and genotypes 1-150-81 and 1-2347-144 did not differ in percent emergence, days to flowering, plant height, mycosphaerella blight score, lodging and days to maturity at all four site years (Table 1). CDC Bronco was higher in mean 1000 seed weight (219 g) than 1-150-81 and 1-2347-144 with 207 and 205 g, respectively. CDC Bronco had the highest grain yield (2.83 t·ha^−1^), significantly greater than genotypes 1-150-81 and 1-2347-144 with 2.36 and 2.33 t·ha^−1^, respectively.

### 2.2. Accumulation of Phosphorus and Phosphorus-Containing Compounds during Seed Development

In seed coat extracts of CDC Bronco, 1-150-81 and 1-2347-144, there was no detectable amount of IP_6_ or other inositol polyphosphates during seed development from 14 to 49 DAF (data not shown [27]). In addition, no lower inositol phosphate intermediates (IP_1_, IP_3_, IP_4_, IP_5_) other than phytic acid were detected in cotyledons of CDC Bronco, 1-150-81, and 1-2347-144 in any of the developmental stages analyzed (data not shown [27]).

**Table 1:** Agronomic traits of pea (*Pisum sativum* L.) cultivar CDC Bronco and low-phytate genotypes 1-150-81 and 1-2347-144 assessed at Saskatoon and Rosthern, Saskatchewan in 2010 and 2011. Values represent means ± SE, *n* = 16. Different letters within a column indicate a significant difference at *p* < 0.05 based on LSD test.

Genotype	Emergence Count (%) ^d^	Days to Flower	Plant Height (cm) ^e^	Mycosphaerella Blight Score (0–9 Scale) ^f^	Lodging Score (1–9 Scale) ^g^	Days to Mature	Grain Yield (t·ha^−1^) ^h^	1000 Seed Weight (g)
CDC Bronco	56 ± 3.0 ^a^	58 ± 1.0 ^a^	79 ± 1.0 ^a^	5.3 ± 0.1 ^a^	5.4 ± 0.2 ^a^	100 ± 1.0 ^a^	2.83 ± 0.1 ^a^	219 ± 2.2 ^a^
1-150-81	55 ± 3.0 ^a^	59 ± 1.0 ^a^	78 ± 2.0 ^a^	5.2 ± 0.1 ^a^	5.3 ± 0.1 ^a^	102 ± 1.0 ^a^	2.36 ± 0.2 ^b^	207 ± 1.6 ^b^
1-2347-144	53 ± 3.0 ^a^	59 ± 1.0 ^a^	76 ± 2.0 ^a^	5.3 ± 0.1 ^a^	5.3 ± 0.1 ^a^	101 ± 1.0 ^a^	2.33 ± 0.2 ^b^	205 ± 2.3 ^b^

^d^ Based on seedlings in a 1 m^2^ section of each plot counted 5 weeks after planting; ^e^ Measured when the pod set was completed; ^f^ Assessed base on 0–9 scale, where 0 = no disease, 9 = completely blighted; ^g^ Assessed based on 1–9 scale, where 1 = erect, 9 = completely lodged; ^h^ Residual harvest weighed after sampling developing seeds (120 pods from each plot).

**Figure 2:**
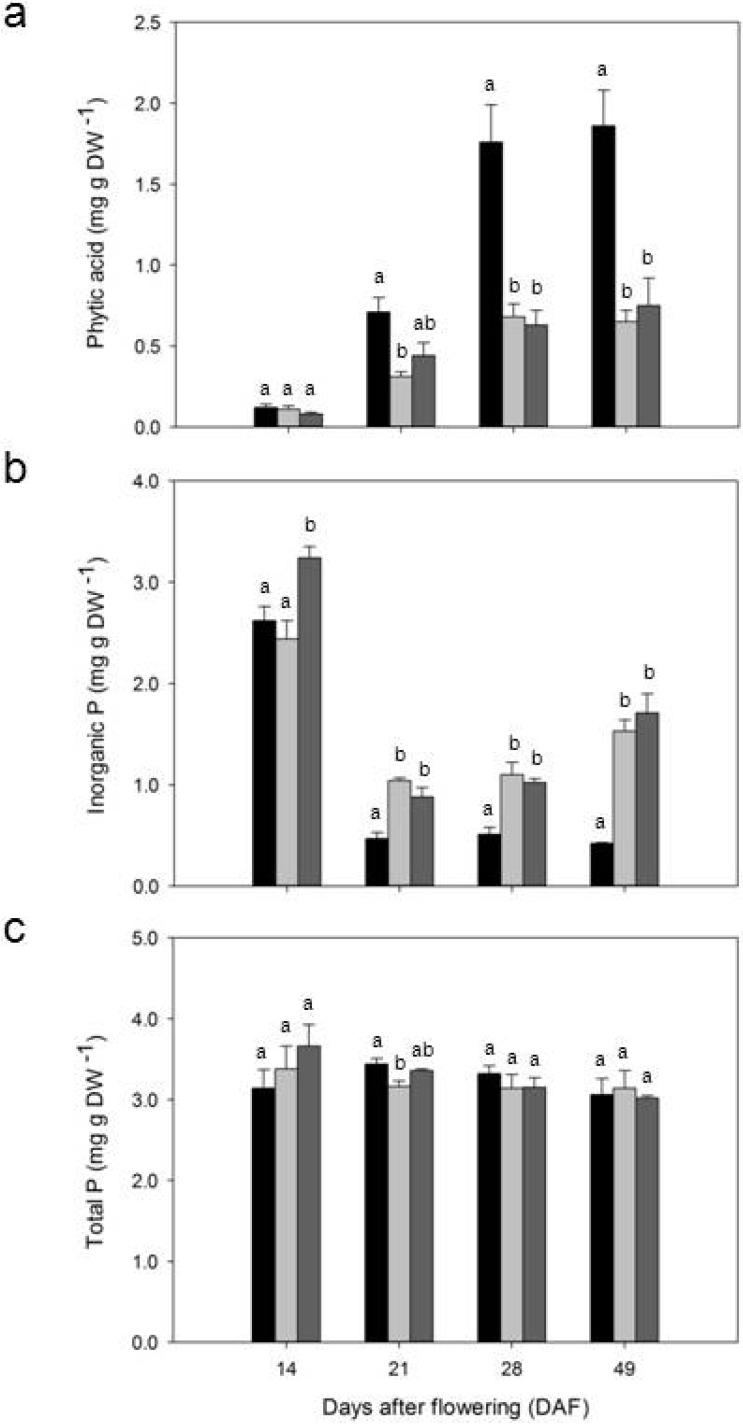
(**a**) Content of phytic acid; (**b**) Content of inorganic P (P_i_); (**c**) Content of total P in developing seeds of pea (*Pisum sativum* L.) for CDC Bronco (black bars) and low-phytate genotypes 1-150-81 (light grey bars) and 1-2347-144 (dark grey bars) assessed at Saskatoon and Rosthern, Saskatchewan in 2010 and 2011. Values represent means ± SE, *n* = 4. Different letters associated with bars within each DAF are significantly different at *p* < 0.05. DAF, days after flowering; DW, dry weight; P, phosphorus.

**Figure A1:**
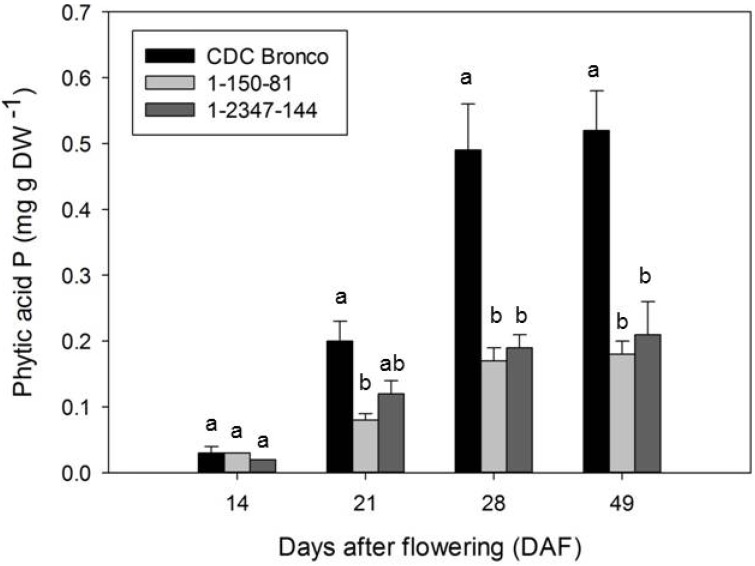
Content of phytic acid P (P_PhA_) in developing seeds of pea (*Pisum sativum* L.) for CDC Bronco (black bars) and low-phytate genotypes 1-150-81 (light grey bars) and 1-2347-144 (dark grey bars). Values represent means ± SE, *n* = 4. Different letters associated with bars within each DAF are significantly different at *p* < 0.05. DAF, days after flowering; DW, dry weight; P, phosphorus P_PhA_, phytic acid phosphorus.

The concentration of IP_6_ at 14 DAF was not significantly different among CDC Bronco, 1-150-81, and 1-2347-144 (Figure 2a). However, the concentrations of IP_6_ among CDC Bronco, 1-150-81 and 1-2347-144 started to differ significantly from 21 DAF onwards (Figure 2a). In CDC Bronco, the concentration of IP_6_ ranged from 0.20 mg·g^−1^ DW at 14 DAF and increased steadily to 1.86 mg·g^−1^ DW at 49 DAF (Figure 2a). A similar trend was observed for IP_6_ in 1-150-81 and 1-2347-144. However, the *lpa* genotypes 1-150-81 and 1-2347-144 showed 65% and 60% reduction in IP_6_, respectively, when compared to their progenitor CDC Bronco at 49 DAF (Figure 2a). In 1-150-81 IP_6_ concentrations ranged from 0.11 to 0.65 mg·g^−1^ DW while the range of IP_6_ concentration in 1-2347-144 was 0.08 to 0.75 mg·g^−1^ DW (Figure 2a). Since it was assumed that all phytic acid-P (P_PhA_) came from IP_6_, these results mirrored those of phytic acid. Absolute values are presented in Figure A1.

P_i_ concentration at 14 DAF was not significantly different for CDC Bronco and 1-150-81 with 2.62 and 2.44 mg·g^−1^ dry weight (DW), respectively (Figure 2b). At 14 DAF, 1-2347-144 had more P_i_ (3.24 mg·g^−1^ DW) than the other two genotypes. From 21 DAF to 49 DAF, P_i_ concentrations between the *lpa* genotypes were similar and significantly higher than CDC Bronco (Figure 2b). At 49 DAF, 1-150-81 and 1-2347-144 were 72% and 84% higher in P_i_, respectively, than CDC Bronco.

The total P accumulation pattern was similar between CDC Bronco and the two *lpa* genotypes (Figure 2c). The concentration of total P was not significantly different between CDC Bronco and the *lpa* genotypes except at 21 DAF. CDC Bronco had 3.44 mg·g^−1^ DW total P at 21 DAF that was significantly different from 1-150-81 and 1-2347-144 with 3.16 and 3.36 mg·g^−1^ DW total P, respectively (Figure 2c). The variations in IP_6_ and P_i_ levels did not affect the total P concentration of these genotypes.

### 2.3. Characterization and Bioinformatic Analyses of PsMIPS

**Figure 3:**
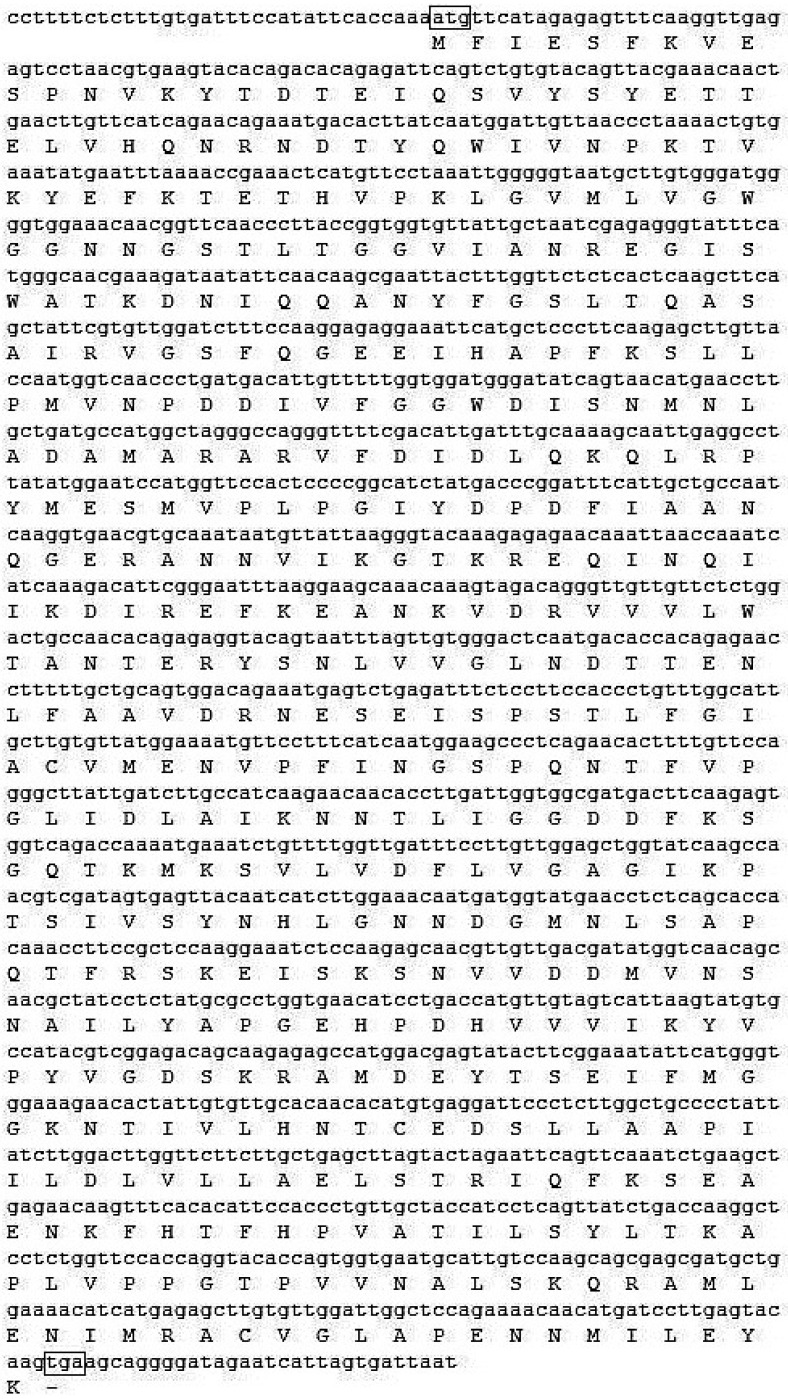
*PsMIPS* cDNA and deduced amino acid sequence from *Pisum sativum* CDC Bronco. Initiation and termination codons are boxed and shown in bold text.

**Figure A2:**
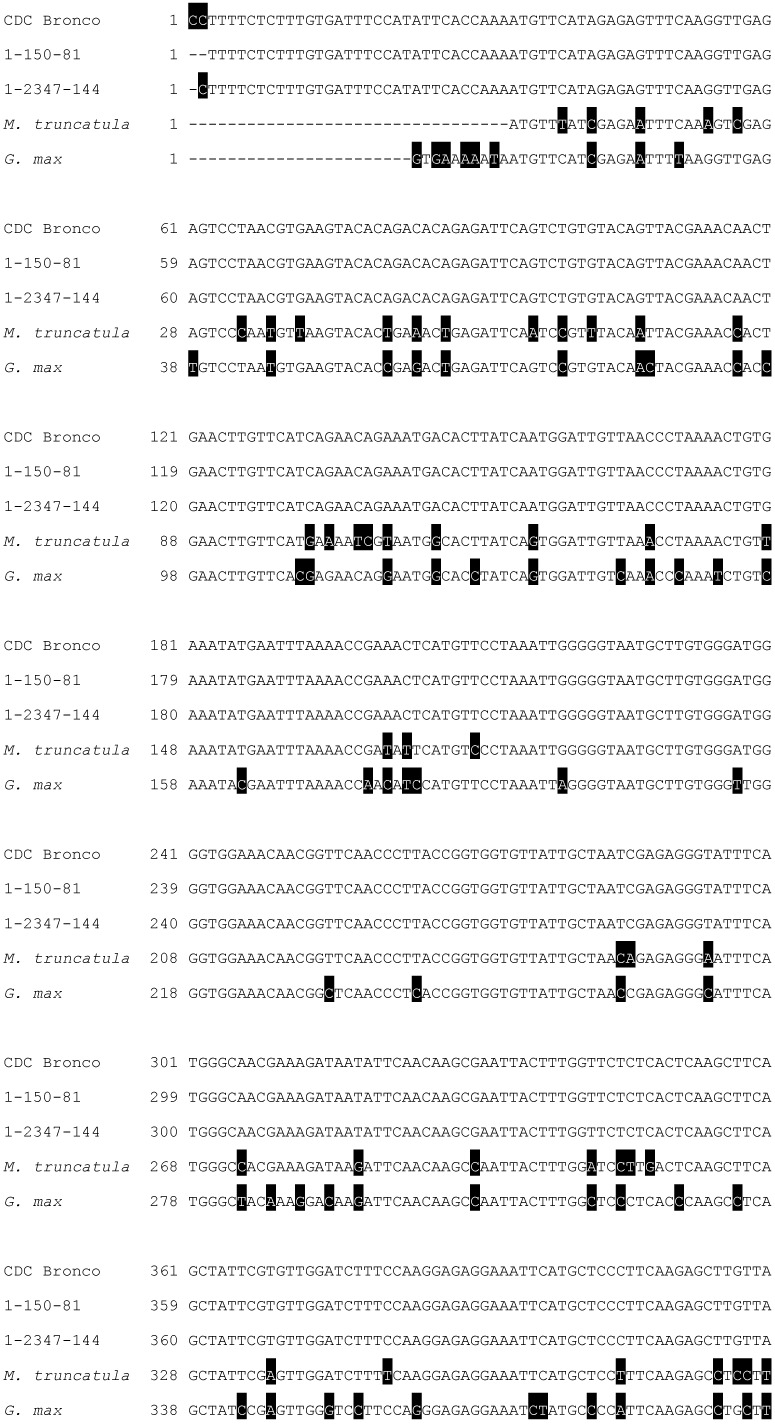

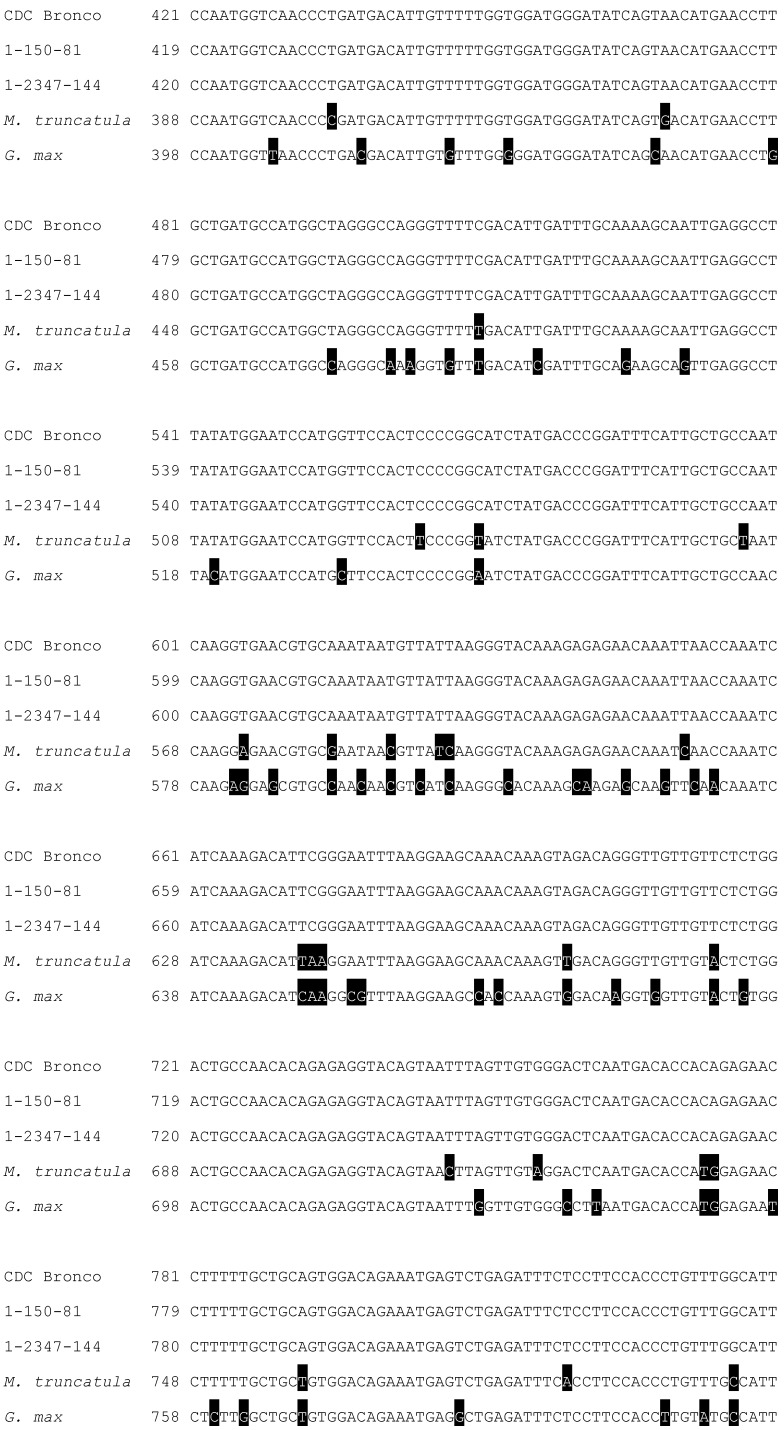

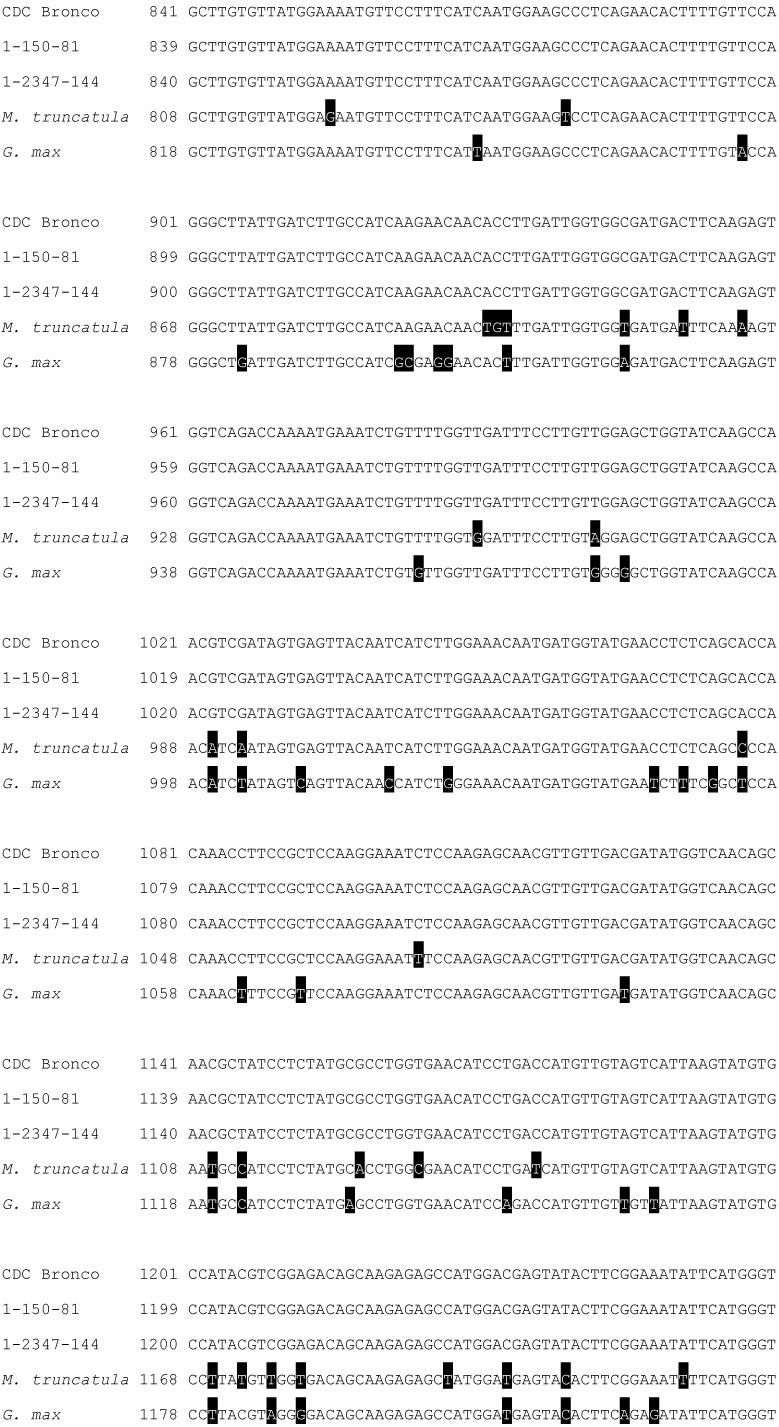

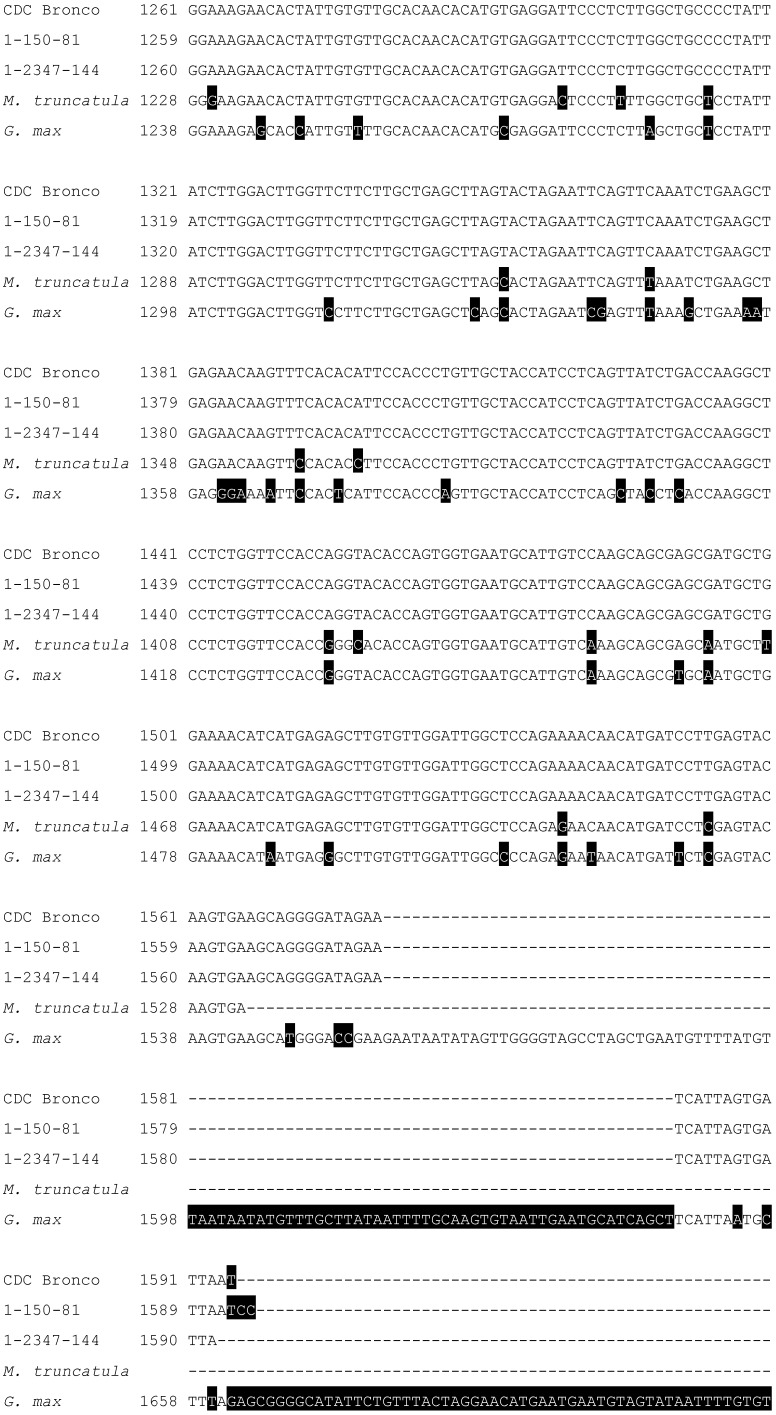

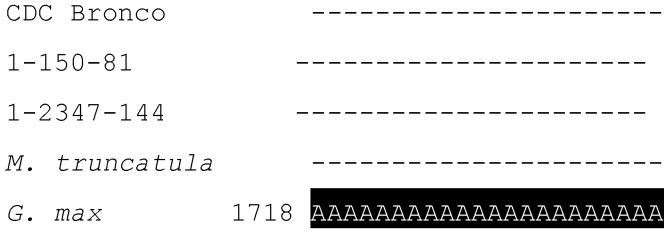
Nucleotide sequence alignment of *PsMIPS* for CDC Bronco and low-phytate genotypes 1-150-81 and 1-2347-144. Also included for comparison are nucleotide sequences for *MIPS* obtained from GenBank for *Medicago truncatula* (XM_003601939.1) and *Glycine max* (AY038802.1). Nucleotide differences are indicated in black boxes.

The *PsMIPS* primers amplified a single product of the expected size (1602-bp) from 14 DAF seed samples of CDC Bronco, 1-150-81, and 1-2347-144 (data not shown [27]). These fragments were excised and sequenced. The obtained cDNA sequence contained a 1530-bp open reading frame (ORF), and encoded a protein of 510 amino acids with a molecular weight of 56.5 kD and pI of 5.35 (Figure 3). The *PsMIPS* ORFs of CDC Bronco and the two *lpa* mutants, 1-150-81 and 1-2347-144, demonstrated a 100% homology (Figure A2). There were no mutations observed in the form of nucleic acid substitutions between the three ORFs.

**Figure 4:**
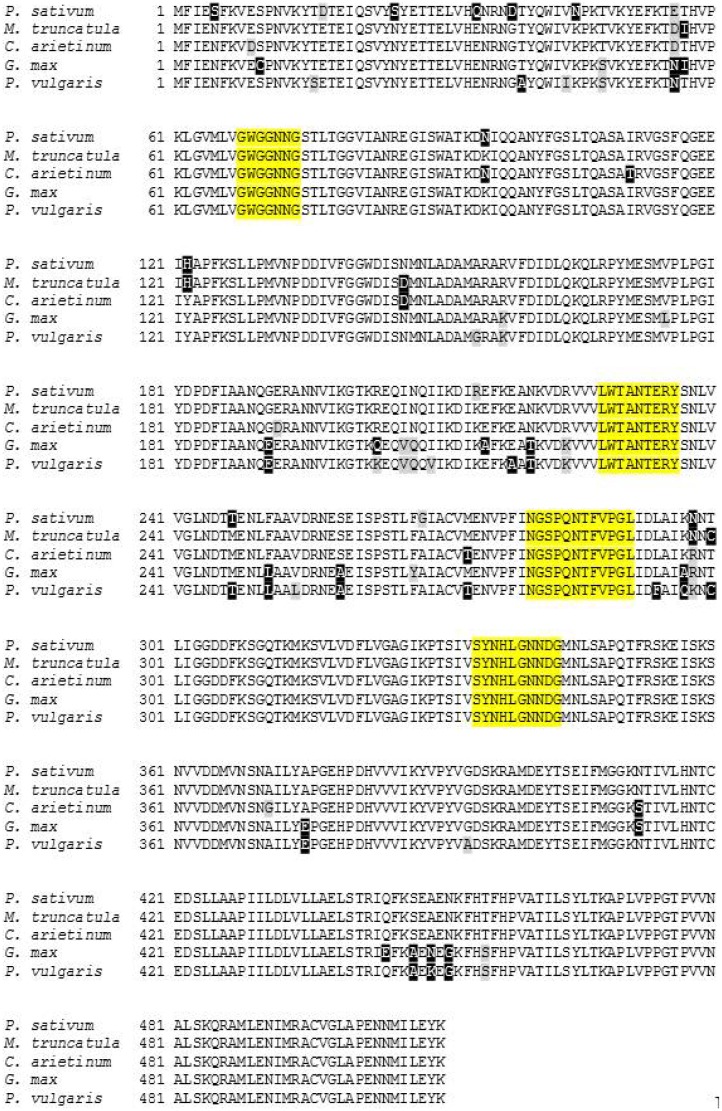
Amino acid sequence alignment of PsMIPS from *Pisum sativum* CDC Bronco with other plant *myo*-inositol phosphate synthase (MIPS) sequences. Conserved domains are highlighted in yellow. Sequences were obtained from GenBank for *Medicago truncatula*, XP_003601987.1; *Cicer arietinum*, NP_001266035.1; *Glycine max*, ABC55420.1; and *Phaseolus vulgaris*, XP_007159720.1. Conserved and unconserved substitutions are indicated in black and grey boxes, respectively.

The deduced amino acid sequence of PsMIPS was aligned along with four other plant MIPS and is shown in Figure 4. The MIPS protein from *Pisum sativum* has a 97% identity with MIPS from *Medicago truncatula*, a 96% identity with *Cicer arietinum*, a 94% identity with *Glycine max* and a 92% identity with MIPS from *Phaseolus vulgaris* (Figure 4). Also present in PsMIPS are four motifs that are highly conserved in all MIPS proteins: GWGGNNG (Domain 1), LWTANTERY (Domain 2), NGSPQNTFVPGL (Domain 3) and SYNHLGNNDG (Domain 4), all of which are involved in cofactor (NAD^+^) binding and reaction catalysis of MIPS protein (Figure 4) [28,29].

**Figure 5:**
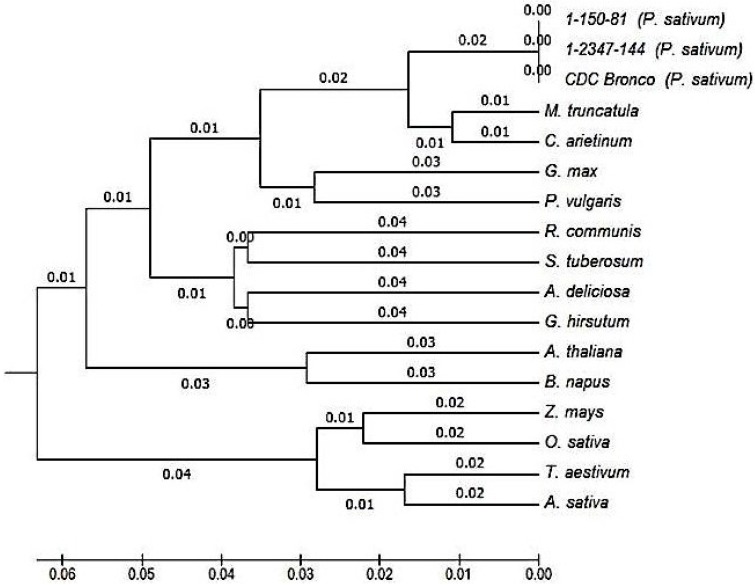
Phylogenetic analysis of MIPS proteins from different plant species including *Pisum sativum* L., CDC Bronco, and low-phytate genotypes 1-150-81 and 1-2347-144. GenBank Accession Numbers include: *Medicago truncatula*, XP_003601987.1; *Glycine max*, ABC55420.1; *Cicer arietinum*, NP_001266035.1; *Phaseolus vulgaris*, XP_007159720.1; *Ricinus communis*, ACU30131.1; *Zea mays*, ACG33827.1; *Oryza sativa*, BAA25729.1; *Triticum aestivum*, AEQ61648.1; *Arabidopsis thaliana*, NP_179812.1; *Actinidia deliciosa*, AFV31635.1; *Brassica napus* ACJ65004.1; *Solanum tuberosum*, XP_006366474.1; *Gossypium hirsutum*, ACJ11714.1; *Avena sativa*, BAB40956.2. The scale bar represents 0.05 substitutions per amino acid site, reflected in the lengths of the branches. Bootstrap values from 1000 iteration analyses are shown in italics.

The PsMIPS protein sequence of CDC Bronco was used in a phylogenetic analysis with 14 other MIPS sequences obtained from the National Center for Biotechnology Information (NCBI) database for a variety of plants. The phylogenetic tree presented in Figure 5 shows the evolutionary divergence among the plant MIPS sequences analyzed. The analysis confirmed PsMIPS protein sequences of CDC Bronco and the two *lpa* genotypes, 1-150-81 and 1-2347-144, to be 100% identical and they clustered together. The monocots, *Z. mays*, *O. sativa*, * T. aestivum*, *and A. sativa* clearly cluster together in one branch. Two distinct sub-branches were obtained for dicots. *P. sativum*, *M. truncatula*, and *C. arietinum* clustered together in a branch, while *G. max* and *P. vulgaris* formed the other branch. MIPS from *R. communis*, *S. tuberosum*, *A. deliciosa*, and *G. hirsutum* were placed in a separate branch. *A. thaliana* and *B. napus* aligned together as an individual branch.

### 2.4. PsMIPS Gene Expression

**Figure 6:**
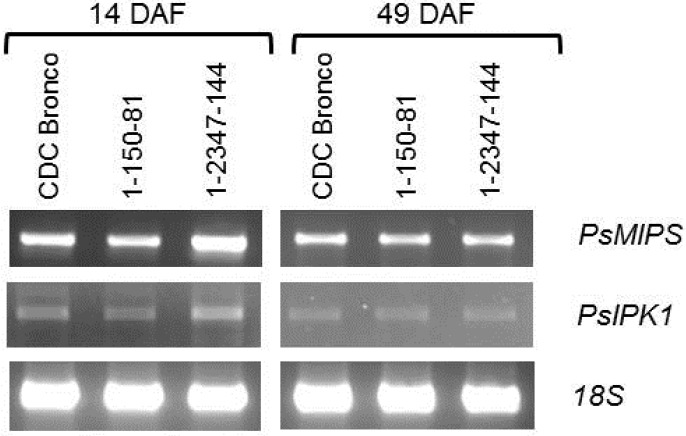
Transcript abundance of *PsMIPS* in developing seeds of pea (*Pisum sativum* L.) for CDC Bronco and low-phytate genotypes (1-150-81 and 1-2347-144) at 14 DAF and 49 DAF as indicated. A fragment of *18S* rRNA used as a loading control is also shown. Results are representative from a minimum of three independent experiments. DAF, days after flowering.

An examination of the *PsMIPS* transcript profile in developing seeds by sqPCR revealed decreased expression levels for CDC Bronco, 1-150-81, and 1-2347-144 at 49 DAF when compared to 14 DAF (Figure 6). In comparison to CDC Bronco, *PsMIPS* expression at 14 DAF was somewhat decreased in 1-150-81 and elevated in 1-2347-144, while levels were essentially the same at 49 DAF (Figure 6).

## 3. Discussion

### 3.1. Agronomic Traits in Low-Phytate Pea

Genes, alleles, and environmental conditions affect the agronomic performance of all crops. It has been demonstrated that *lpa* crops can be generated without major compromises in plant performance [30]. The agronomic performance of 1-150-81 and 1-2347-144 did not significantly differ from CDC Bronco when assessed for traits such as percent emergence, plant height, lodging score, days to flowering, and days to maturity. In soybean *lpa* genotypes, a reduced seedling emergence was observed [31]. The difference in temperature during seed filling was proposed as the reason for the reduced emergence in the low phytate genotypes. Warkentin *et al.* [14] showed that 1-150-81 and 1-2347-144 were slightly different from CDC Bronco in days to flowering and days to maturity. They reported that the 1-150-81 and 1-2347-144 flowered 3 days later and matured 2 to 3 days later than CDC Bronco. This was not observed in the present study. However, the grain yield at maturity was significantly different between 1-150-81, 1-2347-144 and CDC Bronco, with 1-150-81 and 1-2347-144 showing 16% and 17% lower grain yields than CDC Bronco, respectively. This is similar to differences in grain yield observed by Warkentin *et al.* [14]. Since the phytic acid biosynthesis pathway is active in most tissues of a plant, *lpa* mutations can also affect the vegetative processes apart from phytate accumulation [4]. Lower seed weight might be attributed to a reduction in starch accumulation resulting from a defective inositol phosphate synthesis pathway [32]. By targeting a specific gene or its expression to appropriate seed tissues, low-phytate crops can be produced restoring their seed weight and yield [4]. Embryo-specific silencing of expression of an ATP binding cassette (ABC) transporter in maize produced seeds with low-phytic acid with no adverse effect on seed weight [33]. When the myo-inositol methyltransferase (IMT) gene was transferred to *Brassica napus* through a transgenic approach, a 19% to 35% reduction in phytate was achieved without affecting the seed parameters [34]. In addition, rice low-phytate mutants produced through RNAi mediated seed-specific silencing of the inositol pentakisphosphate 2-kinase (IPK1) gene, had no undesirable agronomic characters [35]. Bregitzer and Raboy [36] showed that under irrigated and non-stressful production environments, the barley lpa1-1, lpa2-1, and lpa3-1 produced similar yields as that of the wild type barley. When the low phytate barley cultivar resulting from M640 was evaluated in distinct environments, it had a significantly higher yield when compared with commercial cultivars [37]. These studies provide evidence that low-phytate mutants can be produced in crop varieties without any compromise in agronomic traits.

### 3.2. Redistribution of Phosphorus in Low-Phytate Pea during Seed Development

A large fraction of nutrient P taken up by crop plants is ultimately packaged into seed phytic acid, and this single small molecule represents a major pool in the flux of P in the world’s agricultural ecology. As phytic acid represents a significant portion of total seed P, the accumulation of phytate and inositol phosphates has been studied in different plant species. Grain crops typically contain about 10 mg·g^−1^ phytic acid on a seed dry weight basis, representing about 65 to 85% of seed total P [38,39]. Total P concentration typically ranges from 3 to 4 mg·g^−1^ in seed produced by grain crops, with phytic acid-P (P_PhA_) ranging from 2 to 3 mg·g^−1^.

In this study, the concentration of IP_6_ and its lower isomeric forms (IP_1_, IP_3_, IP_4_ and IP_5_) were analyzed separately in seed coats and cotyledons. There was no traceable amount of inositol phosphates in seed coats. The accumulation of IP_6_ was continuous and linear throughout the stages of seed development and the highest concentration was observed at 49 DAF. The other *myo*-inositol polyphosphates (IP_3_, IP_4_ and IP_5_) and IP_1_ were not present in traceable amounts. In soybean wild-types [40] and maize *lpa1-1*, *lpa2-1* and wild types [10], phytate was reported to accumulate gradually during seed development. P_i_ concentration decreased during seed development in wild type, and total P levels remained relatively consistent. The maize *lpa* genotypes had little to no increase in phytate, and P_i_ concentration was high and did not decrease during development. In barley, the final levels and partitions of P forms are well documented, however it is unclear at what point during seed development the expression of the *lpa* genotype is initiated and how seed P accumulates in developing seeds of different barley *lpa* genotypes [41]. Israel *et al.* [42] compared the changes in seed phytic acid concentrations as well as *myo*-inositol phosphates during seed development between *lpa* and normal phytate genotypes and found that IP_3_ content was relatively low in all genotypes and decreased during seed maturation. The other inositol phosphates such as IP_4_ and IP_5_ were not detected. Larson *et al.* [43] reported that a reduction in seed phytate results in a molar equivalent increase in P_i_ in rice. In the present study, P_i_ concentration in the two *lpa* genotypes was 72% to 84% higher than in CDC Bronco. Between the two *lpa* genotypes, 1-2347-144 had 11% more P_i_ than 1-150-81. Throughout the developing stages, P_i_ concentration was higher at 14 DAF and decreased as IP_6_ accumulation began. Thus, there appears to be an inverse relationship between P_i_ accumulation and phytic acid content. The total P concentration showed no significant difference between CDC Bronco, 1-150-81, and 1-2347-144 except at 21 DAF where CDC Bronco and 1-2347-144 had 8% and 6% more total P, respectively, than 1-150-81. This demonstrates that despite variations in IP_6_ and P_i_ levels, the total P content in the seeds is unaffected. The significantly higher Pi concentration when compared to CDC Bronco in *lpa* pea genotypes shows that the *lpa* genotypes tend to balance the total P levels within the seed to provide adequate amounts of P required for P-related mechanisms in the seed. Unlike the normal genetic and environmental effects that result in quantitative variation in seed total P, *lpa* mutants show large effects on the partitioning of P into phytic acid P and P_i_.

### 3.3. Myo-Inositol-3-Phosphate Synthase (MIPS) Is Identical in Normal and Low-Phytate Pea Genotypes

Genetic mapping and comparison of the position of *lpa* mutation with *MIPS* loci in rice have been reported by Larson *et al.* [43]. They mapped the rice *MIPS* gene on to a locus on chromosome 3, which was orthologous to the *MIPS* gene near maize *lpa1* on chromosome 1S. Previously, *MIPS* gene expression proximal to the site of phytic acid synthesis during grain development in rice was demonstrated by Yoshida *et al.* [44]. Hitz *et al.* [26] confirmed a mutation in *MIPS* responsible for LR33 *lpa* mutation in soybean. Furthermore, higher *MIPS* expression was found in wheat genotypes with high phytic acid levels compared to an *lpa* genotype [45]. These findings have contributed to the interest in *MIPS* as a target for manipulation to produce low phytate crops. *lpa* genotypes of *Arabidopsis*, potato, rice, soybean, and canola have been generated by down-regulation (antisense, RNA interference, or cosuppression) or mutations in *MIPS* gene [24,46,47,48]. Bioinformatic and sqPCR analyses in this study provide evidence that the isolated MIPS gene was specific to phytic acid accumulation in seeds of both the low and normal phytate genotypes. However, the current study has shown that there are no mutations in the *PsMIPS* nucleotide sequences in the *lpa* pea genotypes.

Our sqPCR analysis revealed that at 14 DAF the expression of *PsMIPS* was higher in 1-2347-144 than CDC Bronco and 1-150-81. However, at 49 DAF, its expression was similar in both *lpa* genotypes and CDC Bronco. This further confirms that the reduction in phytate levels caused by *lpa* mutations is not directly controlled by *MIPS* expression. Moreover, mutations affecting *MIPS* are often associated with lower seed yield, seed viability, increased susceptibility to pathogens, and undesired morphology [46,47,49] and, although the seed weight in *lpa* pea genotypes was lower compared to their normal progenitor, the phytate reduction was not detrimental. Therefore, we suggest that the mutation could have occurred in other genes involved in the early stages of the phytate biosynthetic pathway, such as myo-inositol kinases and 2-phosphoglycerate kinase. For example, Shi *et al.* [50] found that the maize *lpa*2 mutant with 30% less phytic acid and three-fold more P_i_ was caused by a mutation in an inositol phosphate kinase gene. Stevenson-Paulik *et al.* [51] generated phytate-free seeds in *Arabidopsis* through disruption of inositol polyphosphate kinases. To gain further insights into the mutation causing the low phytate phenotype in pea, it is necessary to analyze the other enzymes related to phytate biosynthesis. The other possibility is that the mutation could have affected the transport of phytic acid to the vacuole [25]. Mutations in phytic acid ATP-binding cassette transporter reduce the phytic acid content significantly and may also result in absence of lower inositol phosphates [25]. This may possibly explain the fact that there was no lower inositol phosphates detected in this study.

## 4. Experimental Section

### 4.1. Plant Material and Growth Conditions

Seeds of two *lpa* field pea (*Pisum sativum* L.) genotypes (1-150-81 and 1-2347-144; [14], and their progenitor, a normal phytate genotype (CDC Bronco) [52] were obtained from the Crop Development Centre at the University of Saskatchewan. A four-replicate randomized complete block field trial was conducted at two locations in Saskatchewan (Rosthern and Saskatoon) in 2010 and 2011. Field trials were managed using standard techniques for field pea production in Saskatchewan that have been described earlier [14]. Seeding in both years was conducted between May 14 and May 18. Flowers were tagged at the time of flowering and developing seeds were taken 7 days after flowering (DAF) and every 7 d thereafter until maturity on day 49. Pea pods were collected in Ziploc^®^ bags, transported to the laboratory on ice packs and stored at −80 °C until use. Final harvesting in both years was conducted between August 27 and September 17. Field plots were evaluated for several phenotypic parameters during the growing season in each year. These included percent emergence, days to flower, days to maturity, plant height, mycosphaerella blight score, lodging, grain yield and 1000 seed weight. The details of these determinations have been described previously [14].

For molecular studies, seeds of the aforementioned genotypes were sown in 15 cm plastic pots filled with Sunshine^®^ Mix #3/LG3 (Sun Gro Horticulture Canada Ltd., Seba Beach, AB, Canada) and plants grown in a controlled environment chamber (PGR15; Conviron, Winnipeg, MB, Canada) set at 23/18 °C (day/night) temperatures with 16 h day length. The chamber was illuminated with fluorescent lights (T5/HO/835; Sylvania) to provide a photosynthetic photon flux density (PPFD) of 400 μmol photons m^−2^·s^−1^. Plants were regularly provided with a water-soluble fertilizer (Plant-Prod^®^ 20-20-20 Classic; Plant Products Co. Ltd., Brampton, ON, USA) and irrigated with ddH_2_O as required. The experiment consisted of four biological replicates for each genotype. Each replicate consisted of four pots with two plants per pot. Developing seeds were collected at the same time intervals as described above and frozen in liquid nitrogen.

### 4.2. Extraction and Detection of Inositol Phosphates

Seed coats were dissected from the cotyledons and each component was freeze dried (FreeZone 6 Liter Console Freeze Dry System; Labconco, Kansas City, MO, USA) and stored at −80 °C until use. Seed coat or cotyledons were ground using glass beads to a 0.5 mm diameter using a custom designed mill powered with an inverter drive (SM-PLUS Sub-Micro; Leeson Corporation, Grafton, WI, USA). Samples were stored at −20 °C until extraction. Inositol phosphates were extracted from the samples (100 mg) using the extraction method described in [53] with minor modifications [54]. Extracts were filtered using 0.45 µm Acrodisc^®^ syringe filters (25 mm; Pall Corporation, Port Washington, NY, USA) and used immediately for high-performance liquid chromatography (HPLC) analysis.

Detection of inositol phosphates was performed using anion-exchange high performance liquid chromatography (HPLC) on a Dionex ICS 3000 BioLC^®^ system (Dionex, Sunnyvale, CA, USA) and a protocol similar to those reported previously [53,54]. The system consisted of an AS50 Autosampler with a 100 µL injection loop, an AS50 Thermal Compartment (set at 30 °C), a GP50 Gradient Pump and an ED50 Electrochemical Detector coupled with an Anion Self-Regenerating Suppressor (ASRS 300, 4-mm) running in external water mode and a current of 297 mA. Separation was achieved using an OmniPac PAX-100 analytical anion exchange column (Dionex; 8.5 μm, 4 × 250 mm) preceded by an OmniPac PAX-100 guard column (Dionex; 8.5 μm, 4 × 50 mm). Inositol phosphates were separated with a multi-step gradient using water purified by a Milli-Q Water System (Millipore, Milford, MA, USA) to a resistance of ≥18 mΩ. Mobile phases included were water (A), 200 mM NaOH (B), and water/isopropanol (50:50, v/v) (C). The total run time was 80 min, which included an equilibration to starting conditions. A column flow rate of 1.0 mL·min^−1^ was maintained for the mobile phase flow, with a linear gradient profile consisting of solvent A with the following proportions (v/v) of solvent B or C: 0–13 min, B = 6% and C = 12%; 13–30 min, B = 30% and C = 2%; 30–43 min, B = 56% and C = 2%; 43–55 min, B = 56% and C = 2%; 55–65 min, B = 61% and C = 8%; 65–80 min, B = 6% and C = 2%. Chromeleon software (Dionex) was used to plot chromatograms and analyze the data. A standard solution was prepared for each of IP_6_ and its lower isomeric forms IP_1_, IP_3_, IP_4_, IP_5_ (#P8810, #I1267, #I7012, #I5514, #I9261, respectively; all from Sigma-Aldrich, St. Louis, MO, USA). This was used to establish retention times for these compounds, which were subsequently used to determine peak identity in the samples. Inositol phosphate quantification was afforded using external standard curves with *R*^2^ values of 0.99 or greater for each compound. Concentrations for each of the inositol phosphate standards ranged from 1.25 to 25.0 ppm. Phytic acid phosphorus (P_PhA_) was calculated as the number of moles of phytic acid/3.56 as described by [54], assuming all P_PhA_ comes from IP_6_.

### 4.3. Analysis of Phosphorus Levels

Inorganic phosphorus (P_i_) levels were determined as described by [14]. Ground cotyledon samples (50 mg) were extracted overnight at 4 °C in 1 mL of 0.4 M HCl followed by vigorous mixing. A 10 μL aliquot of the extract was aliquoted into a microtiter plate with 90 μL of ddH_2_O and 100 μL of freshly prepared Chen’s reagent. Chen’s reagent contains 6 N H_2_SO_4_, 2.5% (w/v) (NH_4_)_2_MoO_4_ (ammonium molybdate), 10% (w/v) ascorbic acid, and ddH_2_O (1:1:1:2) [54]. The mixtures were incubated for two hours at room temperature before reading the *A*_655_ with a microplate absorbance spectrophotometer (xMark™; Bio-Rad Laboratories, Hercules, CA, USA) against a water blank. Standard curves of K_2_HPO_4_ were constructed ranging from 10 to 50 ppm with *R*^2^ values of 0.95 or greater. Sample values were interpolated from these curves and expressed on a dry weight (DW) basis.

Total P in cotyledons was assayed by the wet ashing method [10]. Ground samples (50 mg) were incubated with 1 mL of concentrated (18.4 M) H_2_SO_4_ overnight at room temperature. Two hundred microliters of 30% (v/v) H_2_O_2_ were added and the samples were incubated in a heating block between 220 and 250 °C for 30 min. Samples were removed and allowed to cool at room temperature for 15 min. This cycle was repeated until the sample became clear. The volume of the samples was adjusted to 6.25 mL with ddH_2_O and total extractable P was determined spectrophotometrically using the method of [55] as described above.

### 4.4. Reverse Transcriptase-Polymerase Chain Reaction (RT-PCR) and Sequencing

Total RNA was isolated from 75 mg of seed from 14 DAF and 49 DAF samples with an RNeasy^®^ Plant Mini Kit (Qiagen, Valencia, CA, USA) using the RLC buffer according to the manufacturer’s instructions. The RNA was eluted in 30 μL of RNase-free water. The samples were quantified (*A*_260_) and purity (*A*_260_:*A*_280_) determined using a Nanodrop 8000 spectrophotometer (Thermo Fisher Scientific, Ottawa, ON, USA). Gel electrophoresis on denaturing 1.2% (w/v) agarose gels containing formaldehyde was used to examine the quality of the RNA. This was assessed by the sharpness of the rRNA bands and 2:1 ratio of 28S rRNA to 18S rRNA. Gels were run in 1× 3-(N-morpholino) propanesulfonic acid (MOPS) buffer and stained with ethidium bromide (Sambrook and Russell 2001). The isolated RNA was stored at −80 °C until further use. cDNA was synthesized from 100 ng of total RNA using the QuantiTect^®^ Reverse Transcription Kit (Qiagen, Valencia, CA, USA) as described by the supplier.

The annotated Cool Season Food Legume Genome Database (http://www.coolseasonfoodlegume.org/) [56] was searched for *MIPS* and a contig in *Pisum sativum* identified (Pisum_sativum_v2_Contig5216) which contained the entire coding region of the *PsMIPS* gene, confirmed by translation and alignment with alfalfa (*Medicago sativa*) *MIPS* (GenBank Accession Number EF408869.1). Gene specific primers for *PsMIPS* were designed from this contig using the Primer-BLAST tool (http://www.ncbi.nlm.nih.gov/tools/primer-blast/) [57] at the National Center for Biotechnology Information (NCBI) to amplify a 1602-bp fragment encompassing the 1530-bp open reading frame (ORF). cDNA was amplified by polymerase chain reaction (PCR) using an iCycler (Bio-Rad Laboratories) and a thermostable DNA polymerase (Q5 High-Fidelity; New England Biolabs, Whitby, ON, USA). Forward (5'-ATGTTCATAGAGAGTTTCAAGGTTGAGAGT-3') and reverse (5'-GCTTGTGTTGGATTGGCTCCAGA-3') primers were used at a final concentration of 0.5 μM each, and 2 μL of the cDNA reaction was used as a template in the 25 μL PCR reaction. The following cycling conditions were used: cDNA denaturation at 98 °C for 30 s, then 35 cycles of denaturation at 98 °C for 10 s, annealing at 55 °C for 30 s, extension at 72 °C for 1 min, followed by a final extension at 72 °C for 2 min. PCR products were visualized on a 1.2% (w/v) agarose gel run in 1× Tris-Acetate-EDTA (TAE) and stained with ethidium bromide [58]. The PCR product corresponding to 1602-bp was excised from the gel and extracted using the QIAPrep Gel Extraction Kit (Qiagen) according to the manufacturer’s instructions. Extractions from multiple PCR reactions were pooled and directly sequenced using the BigDye^®^ Terminator v3.1 Cycle Sequencing Kit (Applied Biosystems, Foster City, CA, USA) at the National Research Council of Canada (NRC; Saskatoon, SK, Canada).

### 4.5. Semi-Quantitative Reverse Transcriptase-Polymerase Chain Reaction (sqRT-PCR)

Transcript levels of *PsMIPS* were examined using sqRT-PCR and performed using the Verso 1-Step RT-PCR ReddyMix™ Kit (Thermo Fisher Scientific, Ottawa, ON, USA) as recommended by the supplier. Targets were amplified using specific primers to *PsMIPS* or pea *18S* small subunit nuclear rRNA. Primers (forward primer, 5'-CATTGGAGGGCAAGTCTGGT-3'; reverse primer, 5'-CCAGCGGAGTCCTAAAAGCA-3') for pea *18S* (GenBank accession number U43011.1) generated a 510-bp amplicon that was used as a reference gene [59]. RNA was isolated as described above and 100 ng was used as template in the 25 µL reactions. The following cycling conditions were used: cDNA synthesis at 50 °C for 15 min, enzyme inactivation at 95 °C for 2 min, then 40 cycles of denaturation at 95 °C for 20 s, annealing at 55 °C for 30 s, extension at 72 °C for 1 min, followed by a final extension at 72 °C for 5 min. PCR products were visualized on a 1.2% (w/v) agarose gel run in 1× TAE and stained with ethidium bromide [58].

### 4.6. Sequence Analysis

Sequencing results were assembled using the BioEdit sequence alignment editor (v7.2.5, http://www.mbio.ncsu.edu/BioEdit/bioedit.html) [60]. Deduced amino acid sequences were obtained using the Translate Tool on the ExPASy SIB Bioinformatics Resource Portal (http://web.expasy.org/translate/). MIPS nucleotide and protein sequences were obtained from GenBank (www.ncbi.nih.nlm.gov) at NCBI and aligned using BLAST (http://blast.ncbi.nlm.nih.gov/Blast.cgi) [61] and CLUSTALW2.1 at the European Bioinformatics Institute (http://www.ebi.ac.uk/Tools/msa/clustalw2/) [62]. Domain analysis of MIPS amino acid sequences was performed using InterProScan (http://www.ebi.ac.uk/interpro/interproscan.html) [63]. Phylogeny analysis was performed using the MEGA6 program (http://www.megasoftware.net/) [64] and available amino acid sequences (NCBI protein sequence database; http://www.ncbi.nlm.nih.gov/nuccore).

### 4.7. Statistics

All statistical analyses were performed using SAS^®^ 9.3 (SAS Institute Inc., Cary, NC, USA). The experimental data presented are mean values from two locations over two years. Levene’s test was conducted to analyze the homogeneity of variance and the results represent means ± standard error (SE), based on four replications. Significant differences were determined by a one-way analysis of variance (ANOVA), *p* < 0.05. Differences among the means were analyzed by a least significant difference (LSD) post-hoc test at *p* < 0.05.

## 5. Conclusions

In conclusion, this study describes the accumulation patterns of phosphorus compounds in developing seeds of two low-phytate pea genotypes in comparison to their progenitor CDC Bronco. Based on the presented evidence, the possibility of a *MIPS* mutation being responsible for the low phytate trait in these pea genotypes is excluded. Interestingly, no accumulation of lower inositol polyphosphates was observed. Once the causative mutation is identified in these low-phytate pea genotypes, it can then be mapped and used in marker-assisted breeding to select low-phytate genotypes.
